# Dual energy for pulmonary vein isolation using dual-energy focal ablation technology integrated with a three-dimensional mapping system: SmartfIRE 3-month results

**DOI:** 10.1093/europace/euae088

**Published:** 2024-05-02

**Authors:** Mattias Duytschaever, Gediminas Račkauskas, Tom De Potter, Jim Hansen, Sebastian Knecht, Thomas Phlips, Johan Vijgen, Daniel Scherr, Gabor Szeplaki, Hugo Van Herendael, Mads Brix Kronborg, Benjamin Berte, Helmut Pürerfellner, Peter Lukac

**Affiliations:** AZ Sint-Jan Hospital, Ruddershove 10, 8000 Bruges, Belgium; Vilnius University Hospital, Santaros Klinikos, Vilnius University, Vilnius, Lithuania; OLV Hospital, Aalst, Belgium; Gentofte Hospital, University of Copenhagen, Gentofte, Denmark; AZ Sint-Jan Hospital, Ruddershove 10, 8000 Bruges, Belgium; Jessa Hospitals, Hasselt, Belgium; Jessa Hospitals, Hasselt, Belgium; Medical University Graz, Graz, Austria; Heart and Vascular Centre, Mater Private Hospital, Dublin, Ireland; Cardiovascular Research Institute, Royal College of Surgeons, Dublin, Ireland; Ziekenhuis Oost-Limburg, Genk, Belgium; Aarhus University Hospital, Aarhus, Denmark; Hirslanden St Anna, Lucerne, Switzerland; Ordensklinikum Linz Elisabethinen, Linz, Austria; Aarhus University Hospital, Aarhus, Denmark

**Keywords:** Pulsed field ablation, Dual energy, Pulmonary vein isolation, Contact force catheter, VISITAG SURPOINT, Focal catheter

## Abstract

**Aims:**

Contact force (CF)–sensing radiofrequency (RF) catheters with an ablation index have shown reproducible outcomes for the treatment of atrial fibrillation (AF) in large multicentre studies. A dual-energy (DE) focal CF catheter to deliver RF and unipolar/biphasic pulsed field ablation (PFA), integrated with a three-dimensional (3D) mapping system, can provide operators with additional flexibility. The SmartfIRE study assessed the safety and efficacy of this novel technology for the treatment of drug-refractory, symptomatic paroxysmal AF. Results at 3 months post-ablation are presented here.

**Methods and results:**

Pulmonary vein isolation (PVI) was performed using a DE focal, irrigated CF-sensing catheter with the recommendation of PFA at posterior/inferior and RF ablation at the anterior/ridge/carina segments. Irrespective of energy, a tag size of 3 mm; an inter-tag distance ≤6 mm; a target index of 550 for anterior, roof, ridge, and carina; and a target index of 400 for posterior and inferior were recommended. Cavotricuspid isthmus ablation was permitted in patients with documented typical atrial flutter. The primary effectiveness endpoint was acute procedural success. The primary safety endpoint was the rate of primary adverse events (PAEs) within 7 days of the procedure. A prespecified patient subset underwent oesophageal endoscopy (EE; 72 h post-procedure), neurological assessment (NA; pre-procedure and discharge), and cardiac computed tomography (CT)/magnetic resonance angiogram (MRA) imaging (pre-procedure and 3 months post-procedure) for additional safety evaluation, and a mandatory remapping procedure (Day 75 ± 15) for PVI durability assessment. Of 149 patients enrolled between February and June 2023, 140 had the study catheter inserted (safety analysis set) and 137 had ablation energy delivered (per-protocol analysis set). The median (Q1/Q3) total procedure and fluoroscopy times were 108.0 (91.0/126.0) and 4.2 (2.3/7.7) min (*n* = 137). The acute procedural success rate was 100%. First-pass isolation was achieved in 89.1% of patients and 96.8% of veins. Cavotricuspid isthmus ablations were successfully performed in 12 patients [pulsed field (PF) only: 6, RF only: 5, and RF/PF: 1]. The PAE rate was 4.4% [6/137 patients; 2 pulmonary vein (PV) stenoses, 2 cardiac tamponades/perforations, 1 stroke, and 1 pericarditis]. No coronary artery spasm was reported. No oesophageal lesion was seen in the EE subset (0/31, 0%). In the NA subset (*n* = 30), microemboli lesions were identified in 2 patients (2/30, 6.7%), both of which were resolved at follow-up; only 1 was symptomatic (silent cerebral lesion, 3.3%). In the CT/MRA subset (*n* = 30), severe PV narrowing (of >70%) was detected in 2 patients (2/30, 6.7%; vein level 2/128, 1.6%), of whom 1 underwent dilatation and stenting and 1 was asymptomatic; both were associated with high index values and a small inter-tag distance. In the PV durability subset (*n* = 30), 100/115 treated PVs (87%) were durably isolated and 18/30 patients (60.0%) had all PVs durably isolated.

**Conclusion:**

A DE focal CF catheter with 3D mapping integration showed a 100% acute success rate with an acceptable safety profile in the treatment of paroxysmal AF. Prespecified 3-month remapping showed notable PVI durability.

**Clinical trial registration:**

ClinicalTrials.gov Identifier: NCT05752487.

## Graphical Abstract

**Graphical Abstract:**
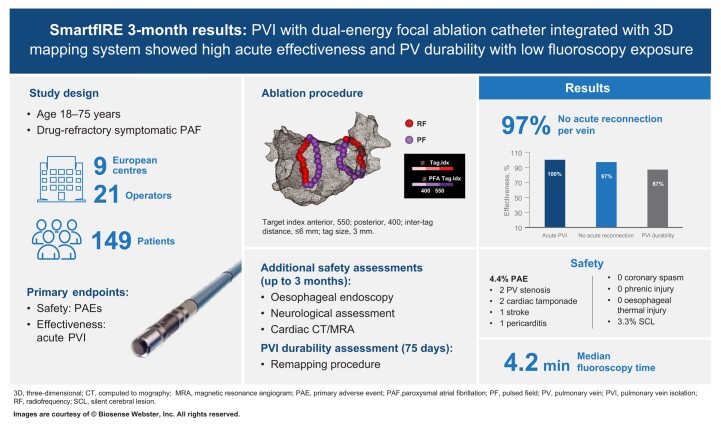

**What’s new?**

- A dual-energy focal contact force catheter that seamlessly delivers radiofrequency and unipolar/biphasic pulse field energy, integrated with a three-dimensional mapping system and pulsed field ablation index, can provide operators with enhanced procedural flexibility on a familiar catheter platform.
- The SmartfIRE study assessed the clinical safety and efficacy of this novel technology in a subset of patients who underwent additional assessments of pulmonary vein (PV) isolation durability, PV narrowing, and oesophageal and neurological safety.
- Results at a 3-month follow-up showed a favourable safety and effectiveness profile of this investigational integrated system.

## Introduction

Atrial fibrillation (AF) is the most sustained common cardiac arrhythmia globally, with increasing rates of incidence and prevalence.^1^ To perform pulmonary vein isolation (PVI), radiofrequency (RF) ablation using point-by-point contact force (CF) catheters has gained widespread user experience. Importantly, clinical evidence from large multicentre studies that investigated CF-enabled RF ablation catheters used with systematic index–guided workflows to create contiguous lesion sets has shown consistently high efficacy and a good safety profile, with low rates of adverse events (AEs).^2–4^ Adoption of these workflows in the real world has shown the reproducibility of outcomes, along with techniques to further minimize fluoroscopy in procedures.^5,6^

Pulsed field ablation (PFA) is a novel, minimally thermal technology used for the ablation of AF that has a preferential action on myocardial tissue through the process of irreversible electroporation.^7^ As reported in large multicentre studies, PFA for PVI showed effectiveness comparable with that of conventional thermal ablation and demonstrated low rates of AEs.^8–10^ Large real-world registry data^11^ showed that procedural-related AEs after PFA were largely attributable to catheter workflow and manipulation, independent of the energy modality.

However, most of the data pertain to large-footprint pulsed field (PF)–only devices, which may be a potential limitation in circumstances such as ablation near conduction tissue^12^ or ganglionic plexi.^13^ Recently, acute renal injury caused by haemolysis has been reported in cases with a large number of PF applications.^14^ The ability to seamlessly switch between RF and PF has the potential to leverage the tissue selectivity of PF energy, especially in situations where there is concern for oesophageal injury, while retaining the ability to use RF for ablation at select locations (e.g. thicker tissue^15^ and near coronary structure^16^) where limitations with PFA may exist. Focal catheter ablation continues to be a critical tool in clinical electrophysiology practice due to widespread user experience and flexibility for lesion sets. Recent pre-clinical studies suggested that both contact and increased CF have an impact on lesion depth with PFA.^17^ These factors led to the development of the CF-sensing dual-energy (DE) THERMOCOOL SMARTTOUCH SF (DE STSF) catheter (Biosense Webster, Inc., Irvine, CA, USA) with multimodality generator TRUPULSE (Biosense Webster, Inc.) to allow operators to combine the tissue selectivity provided by PFA with the proven benefits of RF ablation.

In a randomized pre-clinical study comparing low-dose PFA, high-dose PFA, and RF ablation using this DE catheter, the PF lesion durability assessed by voltage mapping at 28 days was comparable with RF ablation.^18^ On histopathology, the lesion width was similar between the study arms. No collateral damage [phrenic nerve, oesophagus, pulmonary vein (PV) diameter, or peripheral thromboemboli] was observed. In addition, PFA induced more mature scars with benign tissue reaction, while RF ablation was associated with more chronic inflammation and persistence of necrosis.^18^ The SmartfIRE study was conducted to assess the clinical safety and efficacy of this novel DE integrated technology for the treatment of patients with drug-refractory, symptomatic paroxysmal AF. Acute results from up to 3 months of follow-up are presented here.

## Methods

### Study design and patients

The SmartfIRE study (NCT05752487) was an interventional, prospective, multicentre, single-arm study to evaluate the safety and effectiveness of the DE STSF catheter in combination with the TRUPULSE Generator and three-dimensional (3D) mapping system (CARTO; Biosense Webster, Inc.). Eligible patients were aged 18–75 years, were diagnosed with symptomatic paroxysmal AF, had failed or were intolerant to ≥1 antiarrhythmic drug (Classes I–IV), and were clinically indicated for AF ablation by PVI.

The study was reviewed and approved by the ethical committees in all participating sites and by national authorities in the participating countries. The study was conducted in accordance with the International Conference on Harmonization Good Clinical Practices and the Declaration of Helsinki. All enrolled patients provided written informed consent prior to study treatment.

### Ablation system

The DE STSF catheter with the TRUPULSE generator delivers PF applications composed of trains incorporating high-voltage biphasic unipolar pulses of short duration, with each pulse being deployed as a square wave with a positive phase and negative phase separated by a brief delay.^18^ The RF energy ablations were delivered with a target VISITAG SURPOINT index that incorporates CF, power, and time. The PF energy ablations were delivered with a target PF Index^19^ that incorporates the number of PF applications and CF. The CF range was 5–40 g with a recommendation of 10 g.

### Ablation procedure

**Figure 1:**
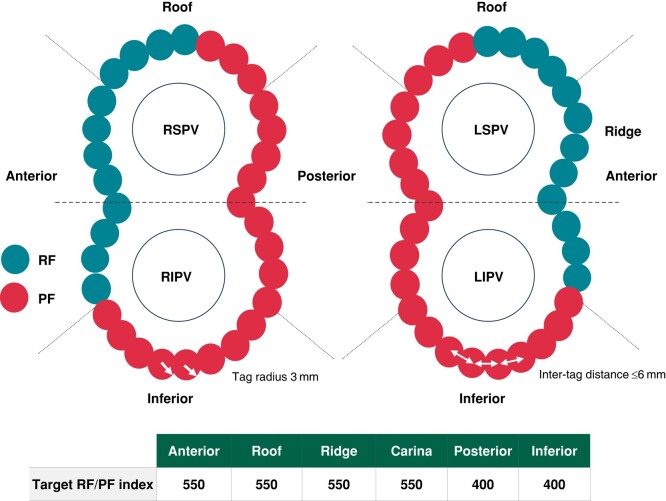
Study ablation workflow for PVI using the dual-energy (RF/PF) contact force catheter. LIPV, left inferior pulmonary vein; LSPV, left superior pulmonary vein; PF, pulsed field; PVI, pulmonary vein isolation; RF, radiofrequency; RIPV, right inferior pulmonary vein; RSPV, right superior pulmonary vein. Images are courtesy of © Biosense Webster, Inc. All rights reserved.

Uninterrupted anticoagulation therapy was administered ≥3 weeks prior to the ablation procedure. Anaesthesia or sedation was delivered per standard laboratory procedure and operator decision. Following transeptal puncture, a left atrial map was created with a high-density mapping catheter (LASSO, PENTARAY, or OCTARAY; Biosense Webster, Inc.). Confirmation of activated clotting time ≥300 s prior to the start of ablation and systemic anticoagulation with heparin was required. Then, PVI was performed using the DE STSF catheter with the recommendation of PFA at posterior/inferior and RF ablation at anterior/ridge/carina segments. Irrespective of energy, a tag size of 3 mm; an inter-tag distance ≤6 mm; a target index of 550 for anterior, roof, ridge, and carina; and a target index of 400 for posterior and inferior were recommended to create a closed contiguous lesion set (*Figure 1* and *Graphical abstract*). PF was recommended at the posterior wall for safety reasons, and RF was recommended for anterior and carina ablation because of its known safety data and its ability to reach tissue thickness in these areas. Pacing of the phrenic nerve was done systematically before and after ablation to assess phrenic nerve injury. Confirmation of PVI (entrance block) was performed after adenosine/isoproterenol and, if necessary, additional applications of PF/RF energy were delivered to treat acute reconnections. Cavotricuspid isthmus (CTI) ablation was permitted with RF or PF with documented typical atrial flutter. If PFA was used near the coronary artery, including for CTI ablation, 1–2 mg intravenous or intracoronary nitroglycerin was recommended. Antiarrhythmic drug management during the study was at the investigator’s discretion.

### Study endpoints

The primary safety endpoint was the incidence of primary AEs (PAEs) within 7 days of the index procedure. The PAEs included major vascular access complication or bleeding, myocardial infarction, pericarditis, pulmonary oedema, stroke or cerebrovascular accident, transient ischaemic attack, thromboembolism, heart block, vagal nerve injury or gastroparesis, cardiac tamponade or perforation (up to 30 days post-procedure), and permanent phrenic nerve paralysis as well as PV stenosis, atrio-oesophageal fistula, and death (up to 90 days post-procedure).

The primary effectiveness endpoint was acute procedural success, defined as electrical isolation of targeted PVs (confirmed by the final entrance block) after adenosine/isoproterenol challenge at the end of the index ablation procedure. The use of a non–study device to achieve PVI was considered an acute procedural failure. Additional effectiveness endpoints included acute reconnection identified by adenosine/isoproterenol challenge. First-pass isolation was defined as the percentage of targeted veins or patients without acute reconnection.

### Prespecified subset safety and pulmonary vein durability assessment

To further delineate safety and assess lesion durability at 2–3 months following ablation, a subset of patients underwent additional PVI durability assessment, cardiac computed tomography (CT) or magnetic resonance angiogram (MRA) imaging for PV narrowing, neurological assessment (NA) for cerebral lesion including silent cerebral lesion (SCL), and an oesophageal endoscopy (EE) in a prospective manner. A repeat electroanatomic map (activation and bipolar voltage) was performed at 75 ± 15 days post–index ablation to verify PVI durability, using the same mapping catheter as was used during the index ablation procedure. A schedule of subset assessment is given in *Table 1*. All imaging procedures performed in the subset were adjudicated by an independent core laboratory.

**Table 1:** Schedule of assessments in a prespecified subset of patients for PV durability and safety

	Pre-procedure (<72 h)	Post-procedure (<72 h)	1 month	3 months
3D electroanatomic remap (*n* = 30)				X^a^
Cardiac CT/MRA (*n* = 30)				X
EE (*n* = 31)		X^b^		
Neurological assessments (*n* = 30)				
Cerebral MRI	X	X	X^c^	X^c^
Neurological examination	X	X	X^c^	X^c^
NIHSS	X	X	X^c^	X^c^
mRS	X		X	X^c^
MMSE	X		X	X^c^

3D, three-dimensional; CT, computed tomography; EE, oesophageal endoscopy MMSE, mini mental state examination; MRA, magnetic resonance angiogram; MRI, magnetic resonance imaging; mRS, modified rankin scale; NIHSS, national institutes of health stroke scale; PV, pulmonary vein.

^a^75 days post-procedure (±15 days).

^b^Between 1 and 3 days (72 h) post-procedure.

^c^Performed if neurological symptoms and/or cerebral ischaemic lesions were identified in a prior evaluation.

### Statistical methods

The safety population analysis set consisted of all enrolled patients who had insertion of the study catheter, regardless of energy delivery. It was used to assess baseline characteristics in this report. The modified intent-to-treat (mITT) analysis set consisted of enrolled patients who met eligibility criteria and had insertion of the study catheter, which is appropriate to assess the occurrence of PAEs in eligible patients. The per-protocol analysis set, used to evaluate the primary effectiveness endpoint, consisted of patients who underwent ablation using PF and/or RF energy via the study ablation system, were treated for the study-related arrhythmia, and had no major protocol deviations that would affect the integrity of the safety and effectiveness data. The NA, EE, cardiac CT/MRA, and PVI durability subset analysis set consisted of patients who provided their informed consent to participate in this subset and complete the necessary additional assessments.

The primary safety endpoint was evaluated using an exact test for a binomial proportion at a one-sided significance level of 2.5%. If the upper bound of the exact two-sided 95% confidence interval of the primary safety endpoint rate was less than the performance goal rate of 12%, the study would be considered to have demonstrated safety. The hypothesis testing was performed in the mITT analysis set.

The primary effectiveness endpoint was evaluated using the exact test for a binomial proportion at a one-sided significance level of 2.5%. If the lower bound of the exact two-sided 95% confidence interval of the primary effectiveness endpoint rate was greater than the performance goal rate of 90%, the study would be considered to have demonstrated effectiveness. The hypothesis testing was performed in the per-protocol analysis set.

The baseline characteristics, safety and effectiveness outcomes, and subset analyses were summarized descriptively. All statistical analyses were performed using SAS 9.4 or SAS Studio 3.8 (SAS Institute Inc., Cary, NC, USA).

## Results

### Patient characteristics

**Figure 2:**
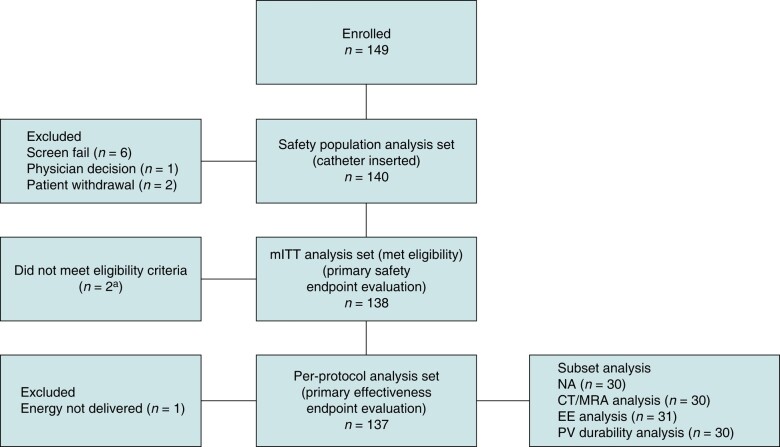
Patient disposition. CT, computed tomography; EE, oesophageal endoscopy; mITT, modified intent-to-treat; MRA, magnetic resonance angiogram; NA, neurological assessment; PV, pulmonary vein. ^a^One patient did not meet the age requirement (18–75 years of age). One patient had some medical problems, and the investigator decided not to include that patient.

Between February and June 2023, a total of 149 patients were enrolled, with 140 constituting the safety population analysis set, 138 constituting the mITT analysis set, and 137 constituting the per-protocol population (*Figure 2*). Baseline characteristics of the safety analysis set are given in *Table 2*; the mean age was 61.6 years, 57.1% of patients were males, and the mean congestive heart failure; hypertension; age ≥75 years (doubled); type 2 diabetes; previous stroke or thromboembolism (doubled); vascular disease; age 65–75 years; and sex category (CHA_2_DS_2_-VASc) score was 1.8.

**Table 2:** Baseline characteristics, safety population analysis set (*n* = 140)

	*n* = 140
Age, mean (SD), years	61.6 (7.8)
Male, *n* (%)	80 (57.1)
CHA_2_DS_2_-VASc score, mean (SD)	1.8 (1.3)
Diagnosis to ablation time, mean (SD), months	53.1 (61.8)
Direct current cardioversion in past 12 months for paroxysmal AF, *n* (%)	34 (24.3)
History of typical right atrial flutter, *n* (%)	13 (9.3)
LVEF, mean (SD), %	56.9 (5.9)
LA diameter, mean (SD), mm	39.5 (5.3)
Failed Class I/III antiarrhythmic drug(s) at baseline, *n* (%)	83 (59.3)
Failed Class II/IV antiarrhythmic drug(s) at baseline, *n* (%)	112 (80.0)
Cardiovascular medical history, *n* (%)	103 (73.6)
Hypertension	76 (54.3)
Coronary disease	22 (15.7)
Congestive heart failure Class II	6 (4.3)
Prior thromboembolic events, *n* (%)	7 (5.0)
Diabetes type 2, *n* (%)	10 (7.1)
Obstructive sleep apnoea, *n* (%)	6 (4.3)

AF, atrial fibrillation; CHA_2_DS_2_-VASc, congestive heart failure; hypertension; age ≥75 years (doubled); type 2 diabetes; previous stroke or thromboembolism (doubled); vascular disease; age 65–75 years; and sex category; LA, left atrial; LVEF, left ventricular ejection fraction; SD, standard deviation.

### Procedural data

Procedural data for the per-protocol analysis set are summarized in *Table 3*. The pre-ablation 3D electroanatomic map was created using a LASSO, PENTARAY, or OCTARAY catheter in 8.0%, 45.3%, and 46.7% of patients, respectively. The median total procedure, DE STSF catheter left atrial dwell, and PV ablation times were 108.0, 77.0, and 54.0 min, respectively. The median fluoroscopy time was short at 4.2 min.

**Table 3:** Procedural information, per-protocol analysis set (*n* = 137)

	*n* = 137
Conscious sedation, *n* (%)	20 (14.6)
General anaesthesia, *n* (%)	117 (85.4)
Total procedure time, median (Q1/Q3), min	108.0 (90.0/126.0)
Catheter type used for LA map, *n* (%)	
LASSO	11 (8.0)
PENTARAY	62 (45.3)
OCTARAY	64 (46.7)
LA mapping time, median (Q1/Q3), min	8.0 (7.0/11.0)
Total fluoroscopy duration, median (Q1/Q3), min	4.2 (2.3/7.7)
Diagnostic fluoroscopy duration	3.2 (1.7/5.6)
Ablation fluoroscopy duration	0.7 (0.2/2.0)
DE STSF catheter LA dwell time, median (Q1/Q3), min	77.0 (64.0/95.0)
Total PV ablation time, median (Q1/Q3), min	54.0 (43.0/67.0)
Total ablation duration, median (Q1/Q3), min^a^	58.0 (43.7/75.0)
Total valid PF/RF application time, median (Q1/Q3), min^a,b^	12.8 (10.0/18.5)
Number of valid PF/RF applications for PVI, median (Q1/Q3)^b^	66.0 (58.0/81.0)
RF; *n* = 136	31.0 (24.0/37.0)
PF; *n* = 137	37.0 (28.0/50.0)
Fluid delivered via the study catheter(s), median (Q1/Q3), mL; *n* = 127^c^	400.0 (300.0/500.0)

CTI, cavotricuspid isthmus; DE STSF, dual-energy THERMOCOOL SMARTTOUCH SF; LA, left atrial; PF, pulsed field; PV, pulmonary vein; PVI, pulmonary vein isolation; RF, radiofrequency.

^a^CTI ablation was included.

^b^The PF ablations with all applications <100% status were excluded and considered as invalid; the time spent on energy delivery was counted as ablation time, excluding the intervals between each delivery.

^c^For 10 patients, fluid was delivered via the study catheter, but the amount was not registered by the site.

Twelve patients underwent CTI ablation (PF only in six, RF only in five, and RF/PF in one patient), with median PF and RF applications of 13.0 and 7.5, respectively. All six patients treated with PF only received pre-treatment of nitroglycerin.

The compliance rate for patient follow-up was 100% at the 7-day, 1-month, and 3-month follow-up visits.

### Safety

A total of 6 PAEs in 6 patients were identified in the mITT analysis set (6/137, 4.4%; *Table 4*). The primary safety endpoint was met with the upper bound of the exact two-sided 95% confidence interval lower than the prespecified performance goal. The PAEs were PV stenosis (two, of which one was a symptomatic event requiring stenting), cardiac tamponade/perforation (two, attributed to difficult transeptal puncture, of which one required pericardiocentesis and the other underwent surgery), stroke (one, recovered at 1-month follow-up without additional treatment and did not induce any sequela), and pericarditis (one, resolved after medications). Per Clinical Events Committee adjudication, all of these PAEs were determined to be procedure related. No coronary artery spasm and no phrenic nerve damage were observed.

**Table 4:** Summary of PAEs, mITT analysis set (*n* = 137)^a^

	*n* (%)
PAEs (≤7 days post-ablation)^b^	6 (4.4)^c^
Atrio-oesophageal fistula	0
Phrenic nerve paralysis (permanent)	0
PV stenosis	2 (1.5)
Cardiac tamponade/perforation	2 (1.5)
Stroke/cerebrovascular accident	1 (0.7)
Transient ischaemic attack	0
Major vascular access complication/bleeding	0
Thromboembolism	0
Myocardial infarction	0
Pericarditis	1 (0.7)
Heart block	0
Pulmonary oedema (respiratory insufficiency)	0
Vagal nerve injury/gastroparesis	0
Death (device or procedure related)	0

mITT, modified intent-to-treat; PAE, primary adverse event; PV, pulmonary vein.

^a^One patient in the mITT analysis set withdrew before the 3-month follow-up and had no PAE, and was thus excluded from the denominator of *n* = 137.

^b^Device- or procedure-related death, PV stenosis, and atrio-oesophageal fistula that occur at 7–90 days and cardiac tamponade/perforation occurring within 30 days post-ablation were also considered as PAEs. Phrenic nerve paralysis was considered a PAE if specified symptoms had not improved at the 3-month visit.

^c^The upper bound of the two-sided exact 95% confidence intervals is 9.3%, less than the prespecified performance goal of 12%.

### Effectiveness

Acute procedural success was achieved in all patients in the per-protocol analysis set (137/137, 100%) and at the level of all PVs separately (533/533, 100%). The primary effectiveness endpoint was met; that is, the lower bound of the exact two-sided 95% confidence interval of 97.3% exceeded the prespecified performance goal. The percentage of targeted veins with acute reconnection identified after adenosine/isoproterenol challenge during index procedure was 3.2%, which converted to be 10.9% of the patients in the per-protocol analysis set. Therefore, the first-pass isolation rate was 96.8% per vein and 89.1% per patient. The acute reconnections detected after adenosine/isoproterenol challenge during the index procedure were distributed across various areas of the encirclement. They were not predominantly noted in either RF- or PF-treated segments.

### Subset analysis

#### Durability rate of pulmonary vein isolation

**Figure 3:**
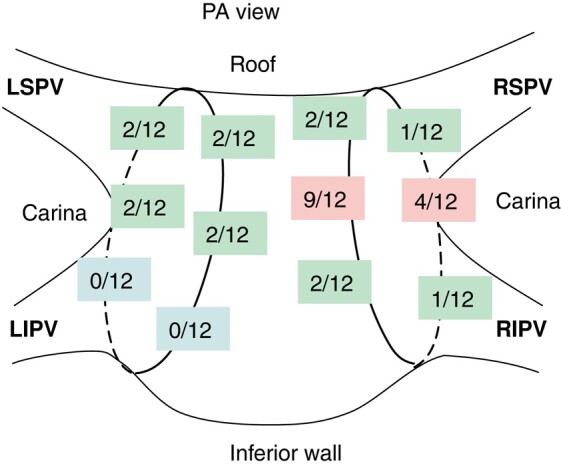
Spatial distribution of reconnections at remapping in patients of PV durability subset. Distribution of reconnections seen in 12 out of 30 subset patients. The posterior carina of the right circle was involved in 9 out of 12 cases. LIPV, left inferior pulmonary vein; LSPV, left superior pulmonary vein; PA, posterior-anterior; PV, pulmonary vein; RIPV, right inferior pulmonary vein; RSPV, right superior pulmonary vein. Images are courtesy of © Biosense Webster, Inc. All rights reserved.

**Figure 4:**
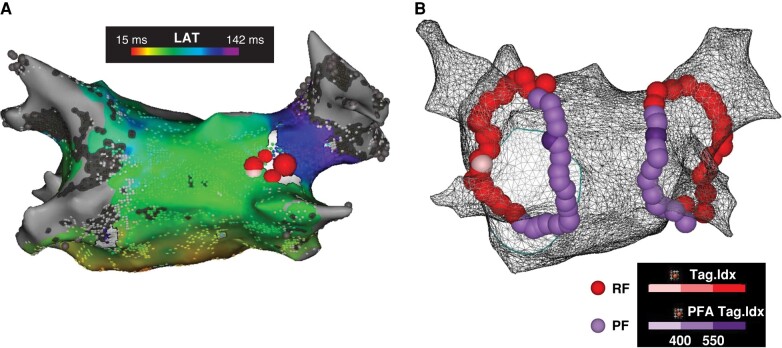
Example of index procedure with remap. Patient from the subset population in which reconnection of the RSPV was observed at the time of the mandatory repeat study. (*A*) Activation map during pacing at the ostium of the coronary sinus during remapping procedure. The reconnection of the RSPV was based on a small gap at the posterior carina of the right circle. (*B*) Ablation set from the index procedure. The contiguity of the circle suggests that non-transmurality of the PF application is the cause of reconnection at the carina. LAT, local activation time; PF, pulsed field; PFA, pulsed field ablation; RF, radiofrequency; RSPV, right superior pulmonary vein. Images are courtesy of © Biosense Webster, Inc. All rights reserved.

A prespecified subset of 30 patients underwent 3D electroanatomic remapping at a mean 79.3 ± 6.9 days post-index procedure, regardless of clinical recurrence status. Durable PVI was verified in 100/115 (87%) veins and 18/30 (60%) patients. At the time of the mandatory remapping study, 3 out of the 12 reconnected patients were symptomatic. The majority (10/12) had reconnection in 1 vein only. The distribution of locations of reconnections is shown in *Figure 3*. In 9 out of the 12 cases, the posterior carina of the right circle was involved. A review of the ablation set in the index procedure was performed to compare it with the location of the reconnection. In 7 out of 12 cases, reconnection was due to discontiguity (greater inter-tag spacing) during the index case; in the other 5 cases, reconnection most likely was due to non-transmurality (lower ablation index; an example is shown in *Figure 4*). In 10/12 patients and in 7/12 patients, the reconnections were seen anteriorly in the RF-treated and posteriorly in the PF-treated sections, respectively. No difference was observed in the incidence of reconnections between the RF- and PF-treated segments. Additionally, the sites of the reconnections seen at remapping did not correspond to the sites of the acute reconnections noted in the index procedure.

#### Assessment via computed tomography/magnetic resonance angiogram

Of the 128 veins among 30 patients in this subset, PV narrowing observed at 3-month CT/MRA was mild (>0–50%) in 85.9% of PVs and moderate (>50–70%) in 3.1% of PVs compared with baseline. Twelve (9.4%) veins had no narrowing. Severe narrowing (>70%) was observed in 2 PVs (2/128, 1.6%, included in PAE; *Table 4*). Of these two, one patient was symptomatic, and dilatation and stenting were performed on the left inferior PV, while the other patient showed left superior PV stenosis without clinical symptoms or intervention. A review of the index procedural ablation data from the 3D navigation system in these two instances showed that multiple overlapping RF lesions leading to excessive ablation with high ablation index values and/or application of lesions inside a small-sized PV may have contributed to the development of the stenosis.

#### Neurological assessment for silent cerebral lesion

A total of 30 patients were included in the NA analysis set. In two patients, a cerebral microembolic lesion located in the parietal lobe was identified by the core laboratory at the discharge visit magnetic resonance imaging (MRI), while no lesions were found at baseline. For the first patient, the lesion was assessed as being symptomatic and adjudicated as PAE (stroke; *Table 4*). The patient reported intermittent stiffness and cramping in the arm starting from Day 1 post–index ablation and lasting 2–3 days. No additional treatment was given. At 1-month follow-up, a repeat scan showed that the lesion had resolved, and the patient had fully recovered. In the second patient, no symptoms were reported (i.e. SCL rate = 3.3%, 1/30), and the lesion resolved on the repeat scan performed at 3-month follow-up. For both patients, there was no neurological sequela; the Mini Mental State Examination (MMSE)–2 score remained unchanged, while Modified Rankin Scale (mRS) and National Institutes of Health Stroke Scale (NIHSS) scores were 0 at baseline and post-ablation.

#### Oesophageal endoscopy

EE was performed at 1–3 days post-ablation in 31 patients. No oesophageal thermal lesions (0/31, 0%) were observed.

## Discussion

The SmartfIRE study demonstrated the safety and acute effectiveness of the DE STSF catheter for the treatment of paroxysmal AF. An acute procedural success rate of 100% was achieved with high first-pass isolation (96.8%), with a PAE rate consistent with other ablation technologies and previous STSF studies (4.4%).^20–22^ Noteworthy is the absence of oesophageal lesions with systematic endoscopy evaluation confirming tissue selectivity of PFA from pre-clinical data. In addition, good PVI durability was demonstrated, with 86% of PVs remaining isolated at scheduled remapping. The acute procedural success rate of 100% and 96.8% first-pass isolation rate per PV demonstrated in the current study compare favourably with those shown in previous studies of PF and RF procedures using ablation index. In the recent ECLIPSE AF study involving 82 patients with paroxysmal or persistent AF in Europe, delivery of optimized PFA with CF-sensing solid-tip focal ablation catheters achieved first-pass isolation in 92.2% of PVs.^20^ A study investigated a focal ablation catheter able to toggle between RF and PF energy in the treatment of 178 patients with paroxysmal or persistent AF and demonstrated first-pass isolation in 95% of PV pairs.^23^ First-pass isolation reported with the STSF RF catheter and index-guided workflow ranges between 73% and 83%.^2–4^

The PAE rate of 4.4% is consistent with past studies of initial experience of novel technologies.^2,3,20,24^ Although PF energy has a promising safety profile for collateral tissue, risk of AEs related to procedure and catheter manipulation are not uncommon.^9,25^ Accurate display of the catheter location and ablation parameters on a 3D navigation system can be beneficial to further minimize procedure-related AEs. It is expected that, with more experience, once the DE catheter platform and integrated system are widely adopted, improvement in safety will be forthcoming. A safety consideration for PFA is the occurrence of coronary artery spasm when ablating near coronary structures.^16^ This has been shown to be mitigated with nitroglycerin pre-treatment.^26^ In our study, of the patients who received CTI ablation, 50% were ablated with PF only and nitroglycerin pre-treatment was given. No evidence of coronary artery spasm was reported, suggesting the feasibility of using either RF or PF for CTI ablation with the DE STSF catheter. PV stenosis is a known severe AE after catheter ablation. Recent AF guidelines reported that the incidence of symptomatic PV stenosis after RF was 0.1–0.8%.^27^ Our study reported both symptomatic and asymptomatic PV stenosis. There continues to be underreporting of PV narrowing and stenosis due to non-specific clinical presentation and a lack of prospective routine post-procedure imaging.^28–30^ In Arentz *et al*.’s study,^31^ 13/47 patients who underwent RF ablation showed PV stenosis, while only 3 of these 13 patients had symptoms. It has been reported that PV narrowing occurs to a greater extent with RF ablation than with PFA.^9,32^ There is a greater association between energy dose dependence and ablation delivered inside the vein.^28^ A pre-clinical study showed that PF energy delivery with the DE STSF catheter and TRUPULSE generator had a wider margin of safety with minimal effect, if any, to PV structure.^18^ These studies are consistent with our observation that RF ablation beyond the recommended guidelines (creating overlap lesions, using a high ablation index, or performing applications inside a small-sized PV) was associated with the PV stenoses. However, further clinical studies comparing RF-only to PF-only ablations using the DE STSF catheter will be necessary to evaluate causality. Cerebral MRI in the current study showed microemboli in 2 patients (2/30, 6.7%), of which 1 was considered SCL (i.e. asymptomatic). Neither patient showed evidence of decreased neurological status based on MMSE, mRS, or NIHSS scores, with the absence of the lesions at follow-up MRI scans. The SCL rate reported in this study is substantially lower than other reported rates with thermal ablation (up to 25%)^33^ or with PFA (7–12%).^8–10,20^ The types of AEs observed in this study are known to be procedure related and not related to PF energy delivery.^8,9,25^ This underlines the importance of continued diligence in catheter manoeuvring during an ablation procedure, even with PF energy.

Utilization of a familiar and standardized ablation procedure may enable improved procedural outcomes.^34,35^ Of note, the total fluoroscopy used in this study was low, with most of it used prior to the start of the ablation, including for transseptal access. The low fluoroscopy is attributed to the real-time visualization of the catheter on the 3D navigation system. Consistent with other published data,^10,23^ PFA devices integrated with a 3D mapping system had lower fluoroscopy times (4.4–9.8 min) compared with PFA catheters that are not fully integrated (21.1–26.0 min).^8,9^ The total RF and PF energy delivery time was <13 min, and the time between the first and last ablations for PVI was 54 min. It is not surprising that the procedural time in this study contrasts to that reported with larger-footprint PF devices. However, it is considerably shorter than many initial experiences with focal catheters.^20,24^ The combined use of PF and RF has resulted in a significant reduction of the ablation time with respect to prior literature describing RF PVI using the CLOSE protocol.^36^ This can be expected to further improve with workflow standardization, similar to the experience with index-guided RF ablation,^6,34,35^ and should be evaluated in a larger population in a real-world setting.^20,23,24^ About 15% of patients (20/137) successfully underwent ablation procedures without general anaesthesia. This percentage is comparable with previous PF studies,^37^ and no significant difference in AEs was observed between patients with general anaesthesia or conscious sedation.

The current report showed a durable PVI in 87% of treated PVs with systematic PV remapping at 2–3 months, providing initial clinical validation of the recommended target index for RF and PF (550 anteriorly, 400 posteriorly). In early studies using both RF/PF and PF-only ablations for PVI, 75% of PVs (58% of patients) were durably isolated.^23^ In a study performing PFA only, PVI durability of 85% per vein and 65% per patient was reported.^38^ In both of these studies, improvement in the PVI durability was seen when the PF waveform was optimized, although a smaller number of patients were in the group. Real-world studies on durability at redo procedures after index PFA reported higher PV reconnection rate per vein, ranging from 29% to 71%.^39,40^ A review of the index procedure ablation set showed that loss of contiguity and non-transmurality were associated with reconnections in all cases, suggesting the importance of workflow in creating a transmural contiguous lesion. Considering the higher variability of tissue thickness in the posterior carina and the proportion of reconnections seen in this region, a higher target index may improve transmurality and should be studied in the future. An additional important parameter in the creation of transmural lesions is catheter stability. A limitation of the study is that in the current software version, catheter stability was not available and PF tags were applied without respiratory gating. Utilizing a future version of the study catheter that incorporates respiratory gating and catheter stability indication and implementation of such a close and optimized workflow may be helpful in the goal of creating contiguous and transmural lesions. This should be studied systematically in future studies to assess the impact on acute and long-term outcomes. While remapping is a good surrogate, 12-month follow-up data will provide clarity on the clinical effectiveness. Thus, conclusions about the effectiveness of the DE STSF ablation system should be based on 12-month results.

A DE focal catheter is clinically relevant in multiple real-world scenarios where leveraging the properties of each type of energy source may be preferable. This DE ablation system provides this flexibility with the ability to seamlessly switch between RF and PF energy delivery while retaining a familiar workflow, thereby offering potential advantages, such as focal catheter design and the capacity to treat different anatomies and areas outside PVI.

### Limitations

The limitations of this study include its single-arm study design. A future study design incorporating RF-only and PF-only cohorts as study arms will be important to address this limitation. The PAE adjudication based on specific energy type (RF or PF) was not an option and would have helped to further understand the relationship between AEs and energy. While the acute PVI and 3-month PVI durability data are positive, 12-month follow-up data are needed to ascertain clinical safety (AEs) and effectiveness (freedom from recurrence) and will be reported in due course. Assessment of PVI durability, as well as additional safety evaluation, was performed in a subset of the overall study population. Specific remapping to evaluate PV reconnections and imaging to measure PV diameter changes should be carried out in a larger population. Additionally, as a new type of technology, the DE STSF ablation system has room for improvement in terms of algorithm and efficiency. Future integration of stability indication with improved technology and workflow may help reduce the PV reconnection.

## Conclusions

Ablation with the DE STSF focal CF catheter with 3D mapping integration shows high first-pass isolation and 100% acute success, with an acceptable safety profile in the treatment of paroxysmal AF. Prespecified 3-month remapping showed notable PVI durability.

## Data Availability

Johnson & Johnson MedTech has an agreement with the Yale Open Data Access (YODA) Project to serve as the independent review panel for the evaluation of requests for clinical study reports and patient-level data from investigators and physicians for scientific research that will advance medical knowledge and public health. Requests for access to the study data can be submitted through the YODA Project site at http://yoda.yale.edu.
